# Distinguishing Healthy and Carcinoma Cell Cultures Using Fluorescence Spectra Decomposition with a Genetic-Algorithm-Based Code

**DOI:** 10.3390/bios13020256

**Published:** 2023-02-11

**Authors:** Marie Pospíšilová, Hana Kalábová, Gabriela Kuncová

**Affiliations:** 1 Faculty of Biomedical Engineering, Czech Technical University, nam. Sitna 3105, 272 01 Kladno, Czech Republic; 2 Institute of Chemical Process Fundamentals of the ASCR, Rozvojova 135, 165 00 Prague, Czech Republic; 3 Faculty of Environment, University of Jan Evangelista Purkyne, Pasteurova 3632/15, 400 96 Usti nad Labem, Czech Republic

**Keywords:** steady state fluorescence, cell suspension auto-fluorescence, genetic algorithm, endogenous fluorophores, cancer biosensor

## Abstract

In this paper, we analysed the steady state fluorescence spectra of cell suspensions containing healthy and carcinoma fibroblast mouse cells, using a genetic-algorithm-spectra-decomposition software (GASpeD). In contrast to other deconvolution algorithms, such as polynomial or linear unmixing software, GASpeD takes into account light scatter. In cell suspensions, light scatter plays an important role as it depends on the number of cells, their size, shape, and coagulation. The measured fluorescence spectra were normalized, smoothed and deconvoluted into four peaks and background. The wavelengths of intensities’ maxima of lipopigments (LR), FAD, and free/bound NAD(P)H (AF/AB) of the deconvoluted spectra matched published data. In deconvoluted spectra at pH = 7, the fluorescence intensities of the AF/AB ratio in healthy cells was always higher in comparison to carcinoma cells. In addition, the AF/AB ratio in healthy and carcinoma cells were influenced differently by changes in pH. In mixtures of healthy and carcinoma cells, AF/AB decreases when more than 13% of carcinoma cells are present. Expensive instrumentation is not required, and the software is user friendly. Due to these attributes, we hope that this study will be a first step in the development of new cancer biosensors and treatments with the use of optical fibers.

## 1. Introduction

The timely diagnosis of cancer improves the chance of successful treatment. Biosensor technology has the potential to detect emerging cancer biomarkers and determine drug effectiveness [1]. Apart from SPR-based biosensors, the most widely used are Fluorescence-based biosensors [2]. For the most part, biosensors of cancer cells are based on the detection of tumour markers which are specific for certain types of cancer cells [3]. In contrast, a biosensor that is based on monitoring the difference in metabolism of healthy and carcinoma cells might be used for detecting various types of cancer.

Cancer cells are involved in energy metabolism that promotes their rapid cell proliferation. The preference for anaerobic glycolysis under normal oxygen conditions is a specific trait of cancer metabolism. Enhanced glycolysis also supports the generation of nucleotides. Increased nicotinamide adenine dinucleotide (NAD) levels enhance glycolysis and fuel cancer cells [4]. Continuous replenishment of NAD promotes the proliferation and survival of fast-dividing cancer cells because elevated NAD levels enhance glycolysis via enzymes that require NAD as a co-enzyme. NAD availability in the cytoplasm is a key determinant of the rate of reduced nicotinamide adenine dinucleotide (NADH) and pyruvate flux to mitochondria and, therefore, the rate of glycolysis [5].

The auto-fluorescent coenzymes NADH and oxidized flavin adenine dinucleotide (FAD) allow label-free detection of cellular metabolism without the need of supplementary analyses to distinguish them from other fluorophores [6,7]. Many studies have documented the ability of autofluorescence analysis to detect early and subtle metabolic changes in cells and tissues, with a promising potential for diagnostic applications [6,8].

The reduced NADH form is fluorescent, while the oxidized NAD+ form is not [9]. In contrast, the oxidized form of FAD is fluorescent, while the reduced form, FADH2+, is not [9]. Since NADH and FAD each represent a different redox state, the quantification of these signals is a useful tool to assess the cell and tissue redox state [9,10]. In measurements of cells and tissues, the fluorescence emissions of NADH and its phosphorylated form NADPH are indistinguishable, so NAD(P)H is often used to represent their combined signals [11].

Non-invasive monitoring of NAD(P)H fluorescence to study metabolic processes was first introduced by Chance [12]. Since that time, NAD(P)H fluorescence has been used for the determination of cell viability [13] and the monitoring of living tissues [14], or the on-line control of biotechnological processes [15,16]. Inside a cell, NAD(P)H exists in two functional forms: free and bound [9,17]. NADH and NADPH bind more than 300 known proteins in cells, including enzymes that are upregulated in cancer [18,19]. The bound forms can be associated with energy generation in the form of ATP, and the relative quantities of free and bound species can provide insight into the metabolic state of the cell. As the absolute amount of NAD(P)H bound to proteins is relatively stable, the ratio of free to protein bound NAD(P)H coincides with the NAD(P) redox state. Simply, a shift in cellular metabolism toward glycolysis and/or lower mitochondrial respiration corresponds with a higher NAD(P)H autofluorescence [20,21].

The protein-bound form of NAD(P)H shows small but detectable spectral differences compared to free NAD(P)H [22]. Autofluorescence of NAD(P)H has been successfully used to differentiate malignant cells from normal cells, which include lung cancer cells, [23] breast cancer cells [24], cervical carcinoma cells [25], leukemic myeloid cells [26], and esophageal epithelium cells [27].

The ratio of free/bound NAD(P)H was measured using many techniques including chemically specific fluorescence quenchers [21], two-photon magnetic resonance, and mainly fluorescence lifetime imaging (FLIM) [28,29,30,31,32]. The high sensitivity of developed FLIM techniques allowed for the determination of the ratio of free/bound NAD(P)H in a cell [33]. Nevertheless, these laboratory methods need expensive instrumentation and are not applicable for on-line measurement. In contrast, steady state fluorescence might be recorded on-line with devices that are inexpensive and might be applied outside the laboratory, where usage of optical fibers for local diagnosis and treatment is technically feasible [34]. In the last decade, progress has demonstrated the great potential of luminescence and absorption-based optical fiber biosensors in the development of cost-effective biomedical devices and point-of-care applications [35].

The fluorescence spectra of living cells are complex as they comprise the fluorescence of fluorescent amino acids from proteins (tryptophan, tyrosine and phenylalanine), cofactors (NAD(P)H, FAD), and vitamins (thiamine, riboflavin pyridoxine). Thus, in these spectra, both frequencies and intensities contain chemical information. The shape of such emission spectra is strongly dependent on the excitation wavelength.

Formerly, complex spectra were deconvoluted with polynomials and defined as Gauss curves [6,36,37] without the incorporation of a linear component of spectra, which characterized variations in homogeneity in samples with living cells. In 2000, J. A. Hageman [38] demonstrated a method of fitting fluorescence spectra with genetic algorithms (GAs). The GAs solve global optimization problems by examining typically hundreds or thousands of trial solutions encoded into data structures called genomes. The algorithm begins with a set of randomly generated genomes defining the first-generation population. The selective exploration of the search space is performed by applying genetic operators to the solutions—randomly combining and perturbing them and choosing the better ones according to the survival-of-the-fittest philosophy. Until now, GAs have been optimized for processing spectral data. A genetic-algorithm-spectra-decomposition software (GASpeD), which combines a genetic algorithm with a traditional local optimization technique, was developed for the analysis of x-ray line spectra emitted by hot dense plasma. The implementation of the population probability variable and preinitiation by starting several partial genetic algorithms further improves the convergence of the GASpeD program and reduces the likelihood of getting stuck at a local minimum [39,40,41,42].

The aim of this work was to analyse the steady state fluorescence spectra of cell suspensions and find differences between healthy and carcinoma fibroblast mouse cells. The task for GASpeD was the deconvolution of the cell suspension spectrum into four peaks related to biofluophores and background. The GASpeD iteration was prolonged to wavelengths of four peaks (lipopigments, FAD, and free/bound NAD(P)H), in agreement with published data.

At a pH of 7.0, the ratio of fluorescent intensities of free/bound NAD(P)H (AF/AB) in healthy cells was always higher in comparison to the ratio in carcinoma cells. In addition, a change of pH in the cell suspension influenced the AB/AF ratio of healthy and carcinoma cells differently. At a pH of 7.0, the AB/AF ratio decreases when a mixture of healthy and carcinoma cells contains more than 13% of carcinoma cells. Expensive instrumentation is not required, and the software is user friendly. Due to these attributes, we hope that this study will be a first step in the development of new label free methods of cancer diagnosis and treatments with the use of optical fibers.

## 2. Materials and Methods

### 2.1. Media

Medium Dulbecco’s Modified Eagle’s (M I) was modified to contain 1000 mg/L glucose, 1 mM sodium pyruvate, and 1500 mg/L sodium bicarbonate (Sigma-Aldrich, Darmstadt, Germany).

Medium (M II) Dulbecco’s Modified Eagle’s (DMEM) was modified to contain 4 mM L glutamine, 4500 mg/L glucose, 1 mM sodium pyruvate, and 1500 mg/L sodium bicarbonate (Sigma-Aldrich, Darmstadt, Germany).

The RPMI-1640 medium was modified to contain 2 mM L-glutamine, 10 mM HEPES, 1 mM sodium pyruvate, 4500 mg/L glucose, and 1500 mg/L sodium bicarbonate (Sigma-Aldrich, Darmstadt, Germany).

The cell samples were diluted in Dulbecco′s Phosphate Buffered Saline (DPBS, Sigma-Aldrich, Darmstadt, Germany, D8537, modified, without calcium chloride and magnesium chloride, osmolality 275,304 mOs/kg). Phosphate buffering maintains the pH at 7.0.

### 2.2. Cell lines and Culture Conditions

All of the in vitro experiments were performed on immortalized BALB/3T3 and CT26.WT mouse fibroblasts or human hepatocellular carcinoma cell line SNU-475. The cell lines were obtained from the American Type Culture Collection (ATCC, Manasas, VA, USA).

Mouse embryo fibroblast BALB/3T3 clone A31 cells (ATCC, Manasas, VA, USA, CCL-163) [43] were cultured as adherent monolayers in plastic Petri dishes Nunc™ (Thermo Fisher Scientific, Waltham, MA, USA) in Dulbecco’s modified eagle medium (Sigma-Aldrich, Darmstadt, Germany), which was supplemented with 10% (*v*/*v*) fetal bovine serum (FBS, Sigma-Aldrich, Darmstadt, Germany) and antibiotic-antimycotic solution (Sigma-Aldrich, Darmstadt, Germany).

The fibroblasts from the colon carcinoma CT26.WT cell line of the mouse [44] were cultured as adherent monolayers in plastic tissue culture dishes with identical conditions. The base medium for this cell line is RPMI-1640 Medium supplemented with FBS and antibiotic-antimycotic solution. Cells from the human hepatocellular carcinoma cell line SNU475 [45] were cultured as monolayers in DMEM (M II) medium supplemented with 10% FBS and antibiotics.

The cells were cultivated up to 70–80% confluence in a Petri dish (100 mm) under normal cell culture conditions (37 °C; 5% CO_2_; 95% humidity) and passaged weekly following the passage protocol [46]. The cell cultures were regularly checked using an inverted phase contrast microscope (Optika XDS-1R, Italy).

For all experiments, cell suspensions were performed from cells cultivated 48 h after passage in a Petri dish. The cultivation medium was drawn off and the dish was washed with 1 mL DPBS twice. Then, a 1.5 mL (0.25% (*w/v*) Trypsin—0.53 mM EDTA solution was added for 8 min in the incubator. It was then checked by microscope to observe if the cells can float freely and 10 mL of culture media was added. The cell suspension was transferred to a cone tube and centrifuged (5 min, 193 g). The supernatant was removed, and the cells re-suspended in 3 mL of DPBS buffer. This cell suspension was poured into a quartz cuvette (volume 5 mL) and the intensity of the fluorescence was measured immediately. PBS buffers with a different pH for measuring fluorescence as a function of environmental pH were prepared by mixing 0.2 M Na_2_HPO_4_•2H_2_O and 0.2 M NaH_2_PO_4_•H_2_O with the same osmolality as DPBS. The mixtures of healthy and carcinoma cell cultures were prepared by the sequential removal of 300 µL from 3 mL of cell suspension in a cuvette filled with 3T3 cell suspension (in DPBS buffer, pH = 7) and replacing them with 300 µL of CT26 cell suspension.

### 2.3. Cell Concentration and Viability

Cell concentrations were determined by microscopy using counting in a Burker chamber. The viability of the cell suspensions was controlled by tryptophan blue staining. Concentration of cells and cultivation medium for each sample is shown in Table 1.

### 2.4. Intensity of Fluorescence (IF)

IFs were measured by a Fluoromax4 HJY [47] with excitation and emission slits of 2 nm. Excitation wavelength was 365 nm and emission spectra were measured in the range of a wavelength of 400–650 nm. Each spectrum run required approximately 45 s.

The data was collected utilizing Fluoromax4 HJY software as a TXT file. The measured intensities of fluorescence of the samples’ *IFs*(*λ*) and buffer *IFb*(*λ*) were corrected by the following calculation:

(1) $$IF{\left(\lambda \right)}_{cor}=IF\left(\lambda \right)*Q\left(\lambda \right)/2.36$$

where *Q*(*λ*) is a detector wavelength quantum sensitivity, and 2.36 is the apparatus constant for the excitation wavelength of 365 nm (Fluoromax4 HJY company data).

The measured data was normalized to eliminate concentration fluctuation by applying:

(2) $$IF_{S}{}_{norm}\left(\lambda \right)=\left[IF_{S}{\left(\lambda \right)}_{cor}-IF_{b}{\left(\lambda \right)}_{cor}\right]/IF_{S}{}_{\max}$$

where *IF_Smax_* is the maximum value from the calculated data [*IFs*(*λ*)*_cor_* − *IFb*(*λ*)*_cor_*].

The normalized data was analysed by GASpeD [40]. The real spectrum was considered with Gaussian-shaped spectral lines and a background continuum approximated by a linear line. Decomposition was performed for four Gaussian curves with FWHM in the range of 0–100 nm and each normalized IF was decomposed in 50 runs. The background was approximated by function:

(3) y=A+kB

***A***, ***B*** are calculated constants and ***k*** is the directivity of the line.

The four Gaussian curves always showed the best fit in comparison to approximation with 3 or 5 Gaussian curves. The best fit was chosen and corrected by Levenberg-Marquardt software to improve that fit. The influence of the buffer pH and the CT26 concentration in the 3T3 sample were analysed in the same way.

## 3. Results

**Figure 1:**
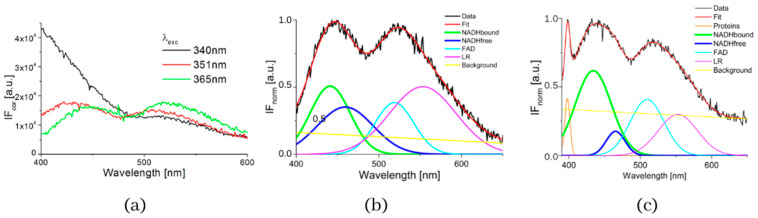
(**a**) Fluorescence spectra of suspension of living human liver carcinoma cells SNU475 (c = 2 × 10^5^ cell/mL) in PBS excited at 340, 351 and 365 nm, (**b**) GASpeD decomposition of the normalized fluorescent spectra excited at 365 nm, (**c**) GASpeD decomposition fluorescence spectra excited at 351 nm.

**Figure 2:**
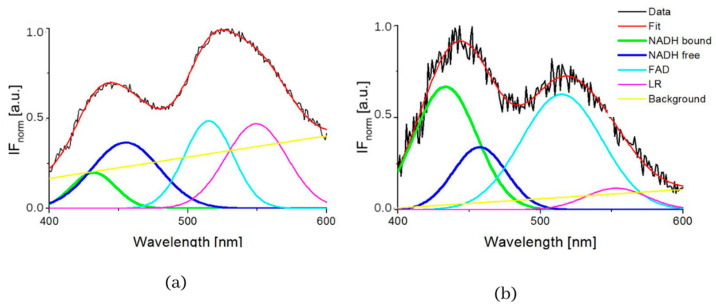
GASpeD decomposition of the normalized fluorescent spectra (**a**) 3T3 sample S4 (healthy cells), (**b**) CT26 sample S1 (carcinoma cells).

The typical measured normalized fluorescence spectra of samples of SNU475 human liver carcinoma cells and the calculated four curves with background by GASpeD are in Figure 1. This figure shows the fluorescence spectra of the cell suspension excited at 340 nm, which is the maximum absorbance of NADH [14]. Excitations of cell suspensions with light of wavelength 351 nm were used in a previous study [28] and, in the case of excitation at 365 nm, the fluorescence of NADH was obviously less influenced by the fluorescence of pyridoxine and tryphophane [6,42]. Fluctuations in measured spectra (black lines) were dependent on the homogeneity of the sample caused by variations in number, shape, and size of the cells. This is reflected in the calculated background (yellow line). We assigned calculated curves as NADH free, NADH bound, FAD and LR (lipofuscin, riboflavin and other lipopigments) [30], because their maxima fitted well with known absorption peaks of these cell components (NADH bound 435, free 460 nm) [6,8,48]. The fluorescence spectra of living cells were strongly dependent on the excitation wavelength as shown in Figure 1a. A change in the excitation wavelength resulted in completely different GASpeD decomposed spectra (Figure 1b,c). In the case of excitation with a wavelength of 365 nm, the best fit, with measured spectrum, was 4 peaks and background. However, for excitation with a wavelength of 351 nm, the best fit was 5 peaks and background. The fifth peak corresponds to the fluorescence of further biofluorophores, mainly pyridoxine and tryptophan. The same results were shown in previous papers [6,49]. In preliminary experiments with pure fluorophores [42], we found that the fluorescence of NADH was minimally influenced by the fluorescence of tryptophan and pyridoxine when excited at 365 nm. Excitations at 365 nm were also used in other fluorescence-based studies on the content of free and bound NADH in living cells [6,42,49]. Typical decomposed normalized fluorescence spectra of healthy 3T3 and carcinoma CT26 cells (excitation at 365 nm) are shown in Figure 2.

**Figure 3:**
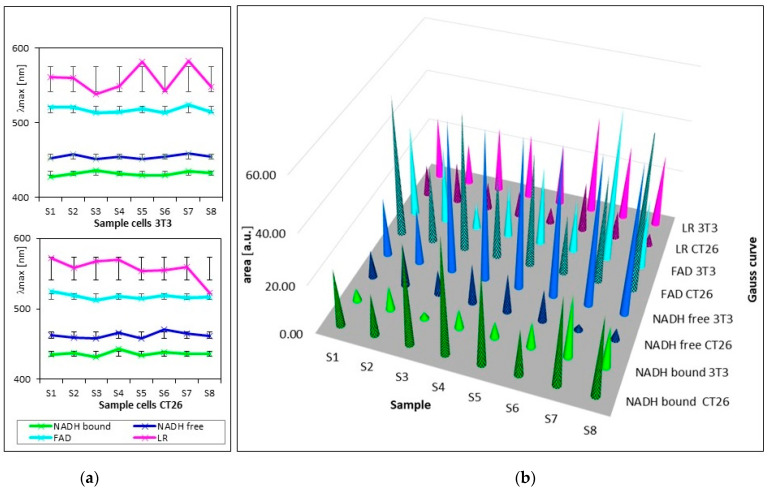
Parameters of calculated curves of deconvoluted spectra of 8 samples of living healthy 3T3 and cancer CT26 cells. (**a**) wavelengths of maxima of calculated Gauss curves (line segments are mean squared deviations σ); (**b**) areas under calculated Gauss curves.

Figure 3 and Table 2 show the summarized calculated parameters of four Gaussian curves for 8 samples of healthy cells (3T3) and 8 samples of carcinoma cells (CT26), measured at pH 7. Wavelengths of maxima (λ_max_) of calculated curves assigned to emission by GASpeD were for NADH bound at 432 ± 2.4 nm, NADH free at 455 ± 2.9 nm, FAD at 518 ± 4.2 nm and LR at 558 ± 17 nm. Deviations from average wavelengths of maxima did not exceed 1% in the case of NADH and FAD. Deviations of four times higher maxima wavelengths of LR relate to the fact that light was emitted by several compounds in this wavelength region, included lipofuscin, riboflavin and lipopigments.

The contents of free and bound NADH were characterized as areas below the calculated Gaussian curves (AB = NADH bound and AF = NADH free). The AF/AB ratios of these areas were from 2 to 25 for 3T3 healthy cells, and from 1 to 0.07 for CT26 carcinoma cells. These ratios are in contrast with previous studies [49], which determined the higher ratio of free/bound NADH in cancer cells rather than in normal cells. This has been explained in terms of the prevalence of anaerobic metabolism in cancer cells. This condition involves fewer stages of interaction between coenzymes and enzymes than in aerobic metabolism. Consequently, the ratio of free/bound NADH is higher in anaerobic cells than in aerobic cells.

**Figure 4:**
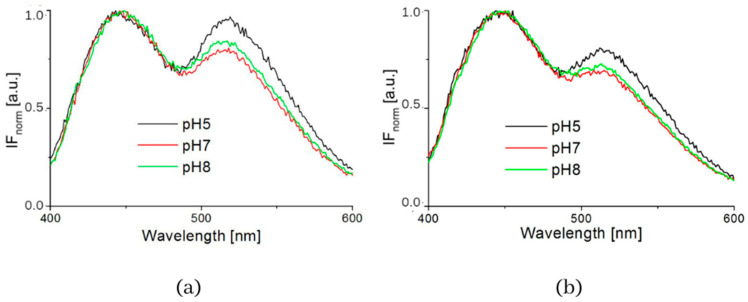
Intensity of fluorescence normalized spectra measured in buffers (pH = 5, 7 and 8) (**a**) 3T3 cells, (**b**) CT26 cells.

**Figure 5:**
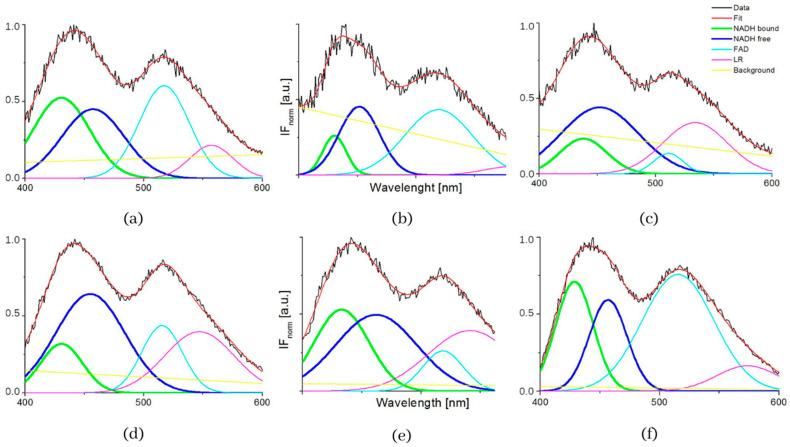
Deconvoluted fluorescence spectra of cells 3T3 and CT26 measured at pH 5, 7 and 8. (**a**) deconvoluted fluorescence spectra of cells 3T3 measured at pH 5, (**b**) deconvoluted fluorescence spectra of cells 3T3 measured at pH 7, (**c**) deconvoluted fluorescence spectra of cells 3T3 measured at pH 8, (**d**) deconvoluted fluorescence spectra of cells CT26 measured at pH 5, (**e**) deconvoluted fluorescence spectra of cells CT26 measured at pH 7, (**f**) deconvoluted fluorescence spectra of cells CT26 measured at pH 8.

The influence of pH was supported by the resulting measurements of NADH fluorescence at pH levels 5, 7 and 8, which are shown in Figure 4. The spectra of both healthy 3T3 cells and carcinoma CT26 cells, as measured in buffers at pH 5 or 8, showed higher peak intensity at 530 nm. The deconvoluted spectra by GASpeD are in Figure 5. The calculated ratios of the areas of free and bound NADH, AF/AB are in Table 3. In contrast to measurements at pH 7, there were less free NADH (AF/AB < 1) in healthy cells at pH 5 than in carcinoma cells (AF/AB > 1). In healthy cells at pH 8 and pH 7, there was a higher level of free NADH (AF/AB > 1). In carcinoma cells, there was minimal free NADH (AF/AB < 1) at pH 8. In deconvoluted spectra measured at pH 8, the most significant differences between healthy and carcinoma cells were in the FAD curves. These results indicate that, for the differentiation of healthy and carcinoma cells based on the ratio of free/bound NADH calculated from fluorescence spectra, it is necessary to keep cultivation conditions identical along with pH during fluorescence measurements.

**Figure 6:**
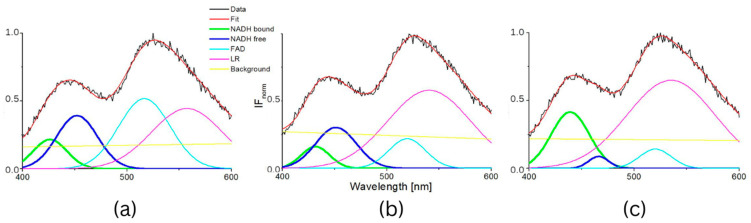
Intensity of normalized fluorescence spectra of suspensions of mixtures of 3T3 cells and CT26 cells (**a**) 4% CT26 (**b**) 13% CT26 (**c**) 27% CT26.

**Figure 7:**
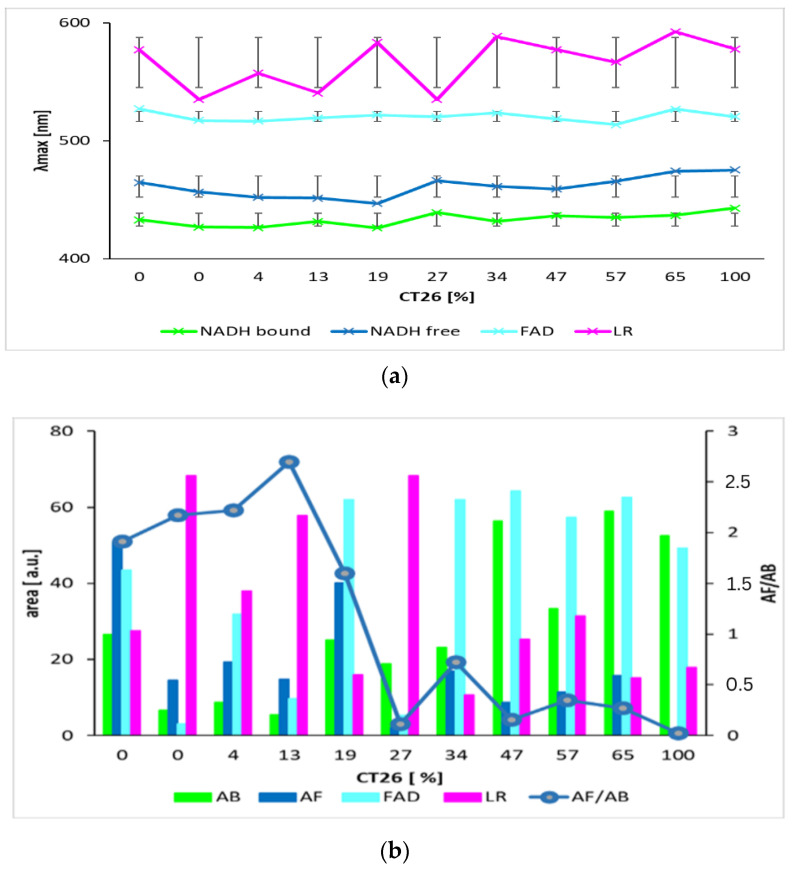
Parameters of calculated curves of deconvoluted spectra of mixtures of living healthy 3T3 and cancer CT26 cells. (**a**) wavelengths of maxima of calculated Gauss curves (line segments are mean squared deviations σ). (**b**) areas under Gauss curves and ratio of bound and free NADH, AF/AB. (The graph shows results of two independent measurements.).

We also applied this proposed method on a mixture of healthy and carcinoma cells. The deconvoluted spectra of mixtures of healthy 3T3 cells and CT26 carcinoma cells measured at pH 7 are in Figure 6 and the calculated parameters are in Figure 7. Wavelengths of maxima of calculated Gauss curves of mixtures were identical as were calculated for pure cell lines. Similarly, they fluctuate within mean square errors regardless of the content of carcinoma and healthy cells. The AF/AB ratio significantly decreases for mixtures with concentrations of carcinoma cells above 13%. 

## 4. Discussion

We measured the steady state fluorescence spectra of suspensions of living cells, healthy cells, and carcinoma mouse embryo fibroblast cells. The excitation wavelength was 365 nm because, in such spectra fluorescence, NADH was minimally influenced by the fluorescence of tryptophan and pyridoxine. Each spectrum was decomposed by GASpeD software into background and four spectra, with one maximum of fluorescence intensity. Wavelengths of excitation and emission light were always shorter than the diameters of cells. Fluorescence (the same way as excitation) light interacted with cells by absorption, excitation and Mie scatter (because the cells’ dimensions were > λ). The intensity of the Mie scatter is a function of the cells’ diameters and cross sections [50]. This scatter is included in the measured fluorescence spectrum. In GASpeD deconvolution, the scatter was calculated as a background. Through this method, the influence of scattered light was eliminated. The deconvoluted peaks should show primary fluorescence, which was emitted by biofluorophores. The calculated wavelengths of maxima of these simple spectra agreed with the published maxima of FAD, bound and free NADH, and lipopigments. In contrast to other deconvolution algorithms, GASpeD considers light scattered like a background (straight line). In cell suspensions, light scatter plays an important role due to variations in the number of cells, cell size, shape, and coagulation. Nevertheless, GASpeD deconvolution did not determine an amount of free and bound NADH, because the software was not able to separate FAD fluorescence caused by absorption of light, at 460 nm, radiated by NADH molecules. Regardless, healthy and carcinoma cells were differentiated according to the intensity of maxima of intensity of free and bound NADH. The deconvoluted spectra of healthy cells have always had a higher maximum of free than bound NADH, the opposite of carcinoma cells. This was valid when cell suspensions were measured at pH 7, however at acidic conditions of pH 5, the AF/AB ratio of healthy cells was lower than that of carcinoma cells. In mixtures of healthy and carcinoma cells, the AF/AB ratio decreases in the presence of more than 13% of carcinoma cells at pH 7. In a previous study [25], the authors measured a higher AF/AB ratio in cancer cells than in normal cells. Our contradictory finding of free/bound NADH ratios, measured at pH 7, might be ascribed to either differences in optimal conditions of live for heathy 3T3 cells, and carcinoma CT26 cells and/or the different impact of conditions of fluorescence measurement e.g., pH on their physiological state. Differences in intensities of autofluorescence of NAD(P)H were used as a sensitive marker for sorting glioma stem cells by flow cytometry [51,52].

NADH autofluorescence is a sensitive marker of cellular redox states and indirectly of cellular energy metabolism. Many recent studies were conducted using fluorescence lifetime imaging microscopy and focused on mitochondria [21]. In contrast, we measured intact cells in suspensions. Microscopy and lifetime measurement are not required, and the software is user friendly. Nevertheless, as other techniques of NADH autofluorescence measurements still requires improvement in both hardware and software, along with more experiments with various cells.

## 5. Conclusions

In this study we revisited a 70 year old method of the measurement of intensity fluorescence spectrum of suspension of living cells in a cuvette. The spectra were normalized, smoothed, and analysed with modified GASpeD [40]. The emission spectra were deconvoluted into four peaks and background. The wavelengths of the peaks matched published data for lipopigments, FAD, and free and bound NAD(P)H. Healthy and carcinoma fibroblast mouse cells were cultivated and measured under identical conditions. At pH 7, ratios of AF/AB for healthy cells were always lower than those of carcinoma cells, which corresponds to the results of previous studies. Changes in pH influenced the AF/AB ratio in different way for healthy cells and for carcinoma cells. In mixtures of healthy and carcinoma cells, the AF/AB ratio decreases when more than 13% of carcinoma cells are present.

We believe that this study has demonstrated a potential of deconvolution of fluorescence spectra with GASpeD for the development of new biosensors and diagnostic methods (non-destructive and without staining) for detecting and recognising carcinoma and healthy cells.

## Figures and Tables

**Table 1:** Cell concentrations in samples S1–S8 (measured at pH = 7).

Samples	S1	S2	S3	S4	S5	S6	S7	S8
Cultivation medium	M I	M II	M II	M II	M II	M II	M II	M II
Concentration 3T3 [cells/mL]	5 × 10^4^	1.25 × 10^5^	5.9 × 10^4^	1.25 × 10^4^	1.17 × 10^5^	5 × 10^4^	2.34 × 10^5^	5 × 10^4^
Concentration CT26 [cells/mL]	5 × 10^4^	1.2 × 10^5^	1.33 × 10^5^	1.37 × 10^5^	5 × 10^4^	1.25 × 10^4^	5 × 10^4^	1.25 × 10^4^

**Table 2:** The ratios of areas under calculated Gauss curves of free and bound NADH, AF/AB at pH 7 in deconvoluted spectra of 8 samples of healthy cells 3T3 and carcinoma cells CT26.

AF/AB	S1	S2	S3	S4	S5	S6	S7	S8	Average	S *
3T3	4.54	2.32	25	2.86	3.03	2.27	1.2	2.27	2.6	7.08
CT26	0.48	1	0.25	0.35	0.43	0.7	0.07	0.15	0.23	0.28

* mean squared deviation.

**Table 3:** The ratios of areas under calculated Gauss curves of free and bound NADH, AF/AB, at pH 5, 7 and 8 in deconvoluted spectra of healthy cells 3T3 and carcinoma cells CT26.

Cells	3T3			CT26		
pH	5	7	8	5	7	8
AF/AB	0.88	3.12	3.33	3.33	0.24	0.84

## Data Availability

The data presented in this study are available on request from the corresponding author.
